# Catheter Ablation for Tachyarrhythmias in Left Ventricular Assist Device Recipients: Clinical Significance and Technical Tips

**DOI:** 10.3390/jcm12227111

**Published:** 2023-11-15

**Authors:** Naoya Kataoka, Teruhiko Imamura

**Affiliations:** Second Department of Internal Medicine, University of Toyama, 2630 Sugitani, Toyama 930-0194, Japan; nkataoka@med.u-toyama.ac.jp

**Keywords:** left ventricular assist devices, atrial fibrillation, ventricular tachycardia

## Abstract

The demand for durable left ventricular assist devices (LVADs) has been increasing worldwide in tandem with the rising population of advanced heart failure patients. Especially in cases of destination therapy, instead of bridges to transplantation, LVADs require a lifelong commitment. With the increase in follow-up periods after implantation and given the lack of donor hearts, the need for managing concomitant tachyarrhythmias has arisen. Atrial and ventricular arrhythmias are documented in approximately 20% to 50% of LVAD recipients during long-term device support, according to previous registries. Atrial arrhythmias, primarily atrial fibrillation, generally exhibit good hemodynamic tolerance; therefore, catheter ablation cannot be easily recommended due to the risk of a residual iatrogenic atrial septal defect that may lead to a right-to-left shunt under durable LVAD supports. The clinical impacts of ventricular arrhythmias, mainly ventricular tachycardia, may vary depending on the time periods following the index implantation. Early occurrence after the operation affects the hospitalization period and mortality; however, the late onset of ventricular tachycardia causes varying prognostic impacts on a case-by-case basis. In cases of hemodynamic instability, catheter ablation utilizing a trans-septal approach is necessary to stabilize hemodynamics. Nonetheless, in some cases originating from the intramural region or the epicardium, procedural failure may occur with the endocardial ablation. Specialized complications associated with the state of LVAD support should be carefully considered when conducting procedures. In LVAD patients, electrophysiologists, circulatory support specialists, and surgeons should collaborate as an integrated team to address the multifaceted issues related to arrhythmia management.

## 1. Introduction

Durable left ventricular assist devices (LVADs) represent potent instruments for enhancing both survival and quality of life for patients afflicted with end-stage heart failure, and the rate of LVAD implantations has increased [1,2]. Tachyarrhythmias originating from both the atrium and ventricle are prevalent in LVAD recipients, akin to patients with advanced heart failure [3,4]. In subjects lacking LVAD support, atrial tachyarrhythmias are typically welltolerated from a hemodynamic perspective. However, ventricular tachyarrhythmias can lead to hemodynamic collapse. Owing to the comprehensive hemodynamic support initially bestowed upon LVAD recipients, concerns related to atrial and ventricular tachyarrhythmias in clinical practice are initially alleviated. Consequently, the impact of refractory tachyarrhythmias on LVAD recipients, which at times precipitates hemodynamic deterioration even under mechanical circulatory supports, remains an unresolved issue. As a result, the management of refractory arrhythmias in LVAD recipients has been a subject of debate.

The current LVADs are electrically actuated continuous-flow pumps represented by the following two devices: the HVAD (Medtronic, Inc., Minneapolis, MN, USA) and the HeartMate 3 (Abbott Labs, Chicago, IL, USA). In patients with a continuous-flow LVAD, in addition to the underlying myocardial dysfunction or scar, the absence of a physiological pulse results in heightened sympathetic nerve activity attributed to the distortion of arterial baroreceptors [5]. This may contribute to an increased incidence of arrhythmias. Furthermore, inappropriately high pump speeds may be predisposed to suction events or ventricular septal migration, resulting in ventricular arrhythmias [6]. In contemporary practice, the utilization of LVADs serves two primary purposes: bridging to transplantation or serving as destination therapy [7]. Since the long-term use of LVAD is known to be associated with an increased risk of arrhythmias, especially in destination therapy recipients, the management of tachyarrhythmias is crucial for the extended use of LVADs [8].

Anti-arrhythmic drugs are recognized as one of the treatment options for tachyarrhythmias. Nevertheless, their negative inotropic and chronotropic effects can pose challenges for heart failure patients with reduced systolic function. Consequently, amiodarone spontaneously emerges as the sole choice for LVAD recipients. However, concerns have emerged regarding the use of amiodarone [9,10]. Therefore, catheter ablation for tachyarrhythmias has emerged as an important strategy. In this paper, we have conducted a literature review on arrhythmias in LVAD recipients and have proposed optimal practices in anticipation of the upcoming heart failure “pandemic” [11].

## 2. Significance of Tachyarrhythmias in LVAD Recipients

### 2.1. Atrial Arrhythmias

In patients prior to LVAD implantation, the incidence of atrial arrhythmias, with the majority being atrial fibrillation (AF), has been reported to range from 21% to 54% in the literature [12]. Additionally, following device implantation, approximately 20% to 30% of patients developed new-onset atrial arrhythmias [12]. In patients without LVAD support, AF is linked to an increased risk of thromboembolism, necessitating anticoagulant therapy, and ventricular dysfunction due to the lack of atrial kick. This may warrant catheter ablation for rhythm control, even in cases of end-stage systolic dysfunction [13].

However, LVAD therapy originally requires warfarin administration to prevent pump thrombosis and ultimately reduce the load on the left ventricle, irrespective of the presence of AF, potentially mitigating the negative impact of AF. Nevertheless, several observational studies have reported that preoperative AF, particularly the persistent type, is associated with increased mortality, a higher degree of functional tricuspid regurgitation, a higher incidence of new-onset ventricular arrhythmias, and a greater occurrence of late right heart failure when compared to individuals without AF [3,14,15,16].

Concomitant left atrial appendage occlusion in patients receiving LVAD implantation may have the potential to reduce adverse events such as stroke or thromboembolisms [17]. On the other hand, registry data have demonstrated that AF is not an independent predictor of stroke in LVAD recipients [3,18]. A systematic review did not demonstrate statistically significant favorable outcomes in LVAD patients receiving concomitant left atrial appendage occlusion [19]. These data suggest that AF in LVAD recipients is not an offender but rather a manifestation of advanced heart failure. Furthermore, there is a lack of clinical data available to guide decision-making for reducing the risk of thromboembolism in LVAD recipients with AF [20].

**Figure 1:**
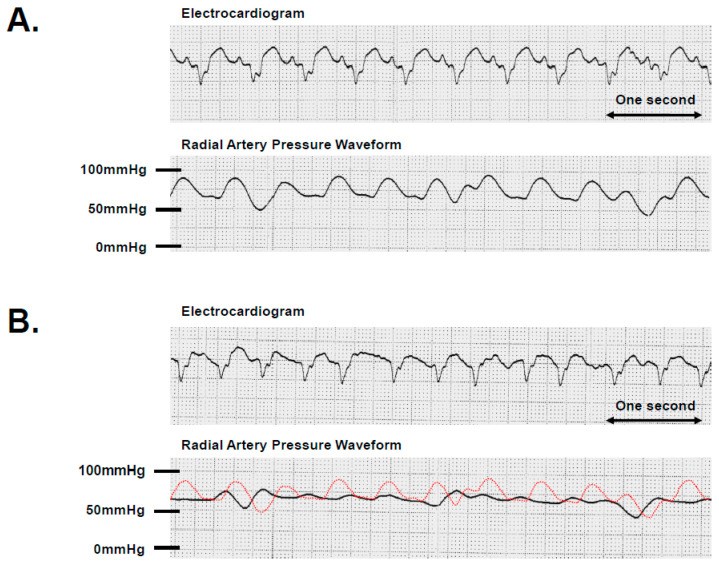
Changes in radial artery pressure with alterations in supraventricular rhythm. A patient with LVAD support exhibited paroxysmal atrial fibrillation shortly after implantation. (**A**) A monitoring electrocardiogram showed 115 beats per minute with a sinus rhythm. Simultaneously, the radial artery pressure displayed a pulsatile waveform. (**B**) A monitoring electrocardiogram displayed 120 beats per minute with atrial fibrillation. The radial artery pressure exhibited a continuous waveform at a level equivalent to diastolic pressure with the disappearance of systolic pressure. She required hemodynamic support with inotropes despite LVAD support during atrial fibrillation. A red dot curve depicts the radial artery pressure waveform in sinus rhythm, identical to that of (**A**).

However, in some cases, hemodynamic instability occurs due to AF, leading to the absence of the atrial kick (Figure 1). If a rapid ventricular response is the sole risk factor for intolerable hemodynamics, atrioventricular nodal ablation with ventricular pacing may represent a potential solution for LVAD recipients. Nevertheless, it is important to note that an animal model supported by a continuous-flow LVAD illustrated the necessity of atrial systolic function in preserving hemodynamic stability during ventricular fibrillation (VF) [21]. The LVAD effectively provided support for 60 min during VF with sinus rhythm; however, it was noted that AF was unable to sustain both systemic and pulmonary circulation in the presence of elevated central venous pressure [21]. This result may, in part, be influenced by right ventricular failure associated with AF; nonetheless, it underscores the significance of maintaining sinus atrial rhythm in such a specific scenario [16,22]. Further studies are warranted to better clarify the clinical implication of the presence of AF during durable LVAD supports.

### 2.2. Ventricular Arrhythmias

Ventricular tachyarrhythmias are common for LVAD recipients, ranging from 20% to 50% depending on the underlying type of cardiomyopathy, existence of preoperative ventricular arrhythmias, and length of follow-up [23,24,25,26]. An observational study reported that a new onset of monomorphic ventricular tachycardia (VT) occurred in 18% of LVAD subjects, in contrast to polymorphic VT or VF [27]. This may be influenced by electrophysiological reverse remodeling following LVAD implantation. The QT interval in surface electrocardiograms, which is associated with polymorphic VT or VF, decreases following implantation due to the unloading of the left ventricle. However, in some cases, there is an increase in fragmentation within the QRS complex, which is associated with fibrosis in the myocardium, and this increase is linked to monomorphic VT [28]. The age at the index implantation and gender differences had no impact on the incidence of ventricular arrhythmias [29,30].

Ventricular arrhythmias occur more frequently in the early postoperative period; therefore, ventricular tachyarrhythmias following LVAD implantation are typically categorized into two phases: early onset within 30 days of surgery and late-onset thereafter [31]. Early ventricular arrhythmias are associated with postoperative unstable hemodynamics, proarrhythmic effects of inotropic agents, electrolyte imbalances, and the suction effect of the pump [31]. The VT-LVAD score, comprising the presence of ventricular arrhythmias prior to LVAD implantation, the absence of angiotensin-converting enzyme inhibitors post-LVAD implantation, the duration of heart failure, ventricular arrhythmias occurring early after LVAD implantation (within <30 days), AF prior to LVAD implantation, and the etiology of idiopathic dilated cardiomyopathy, has been proposed as a risk calculator for predicting the late onset of ventricular arrhythmias [32,33].

Early ventricular arrhythmias have emerged as a significant risk factor for mortality, particularly in patients undergoing destination therapy or experiencing electrical storms of ventricular arrhythmias [34,35,36]. In particular, the occurrence of ventricular arrhythmias during the index hospitalization for HeartMate 3 implantation is associated with a mortality rate approximately twice as high as that in patients without such arrhythmias [4].

However, the clinical impact of late-onset ventricular arrhythmias is still a matter of debate. An observational multicenter registry revealed no statistically significant prognostic discrepancy in the presence of late-onset ventricular arrhythmias when comparing groups with and without such arrhythmias [33]. On the contrary, several previous studies have demonstrated the necessity of converting to sinus rhythm in cases of ventricular tachyarrhythmias [37,38,39,40]. The primary reasons for sinus rhythm conversion are complaints of palpitations or pump flow reduction due to right heart failure. These results may suggest that late-onset ventricular tachyarrhythmias have clinical impacts as contributors to the patient’s quality of life rather than predictors of mortality.

One important point to remember is that ventricular arrhythmias are also associated with right ventricular failure, which, in turn, is linked to mortality [41,42]. Aortic insufficiency is also influenced by ventricular tachyarrhythmias, as the aortic transvalvular gradient persists during VT or VF, in contrast to sinus rhythm, where the gradient disappears during systolic periods [43]. Further investigations to determine the predictive factors for right heart failure in patients with late-onset ventricular arrhythmias are warranted. The burden of VT or VF may be a key factor for discriminating the high-risk group for prognosis, similar to AF in subjects with advanced systolic dysfunction [44]. Concerns regarding the potential for thrombosis in the fibrillating right ventricle also persist [45].

## 3. Catheter Ablation in LVAD Recipients

### 3.1. Technical Tips

**Figure 2:**
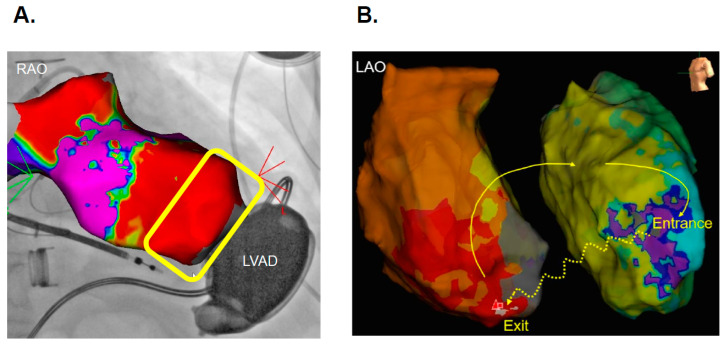
Electromagnetic interference between magnetic field sensors and HVAD. (**A**) The bipolar voltage map within the left ventricle. The presence of electromagnetic interference in the vicinity of the cannula of the HVAD is depicted as the red area enclosed within the yellow line. Local electrophysiological data could not be obtained within the interference area. (**B**) Activation mapping during ventricular tachycardia was conducted using the impedance-based three-dimensional mapping system.

**Figure 3:**
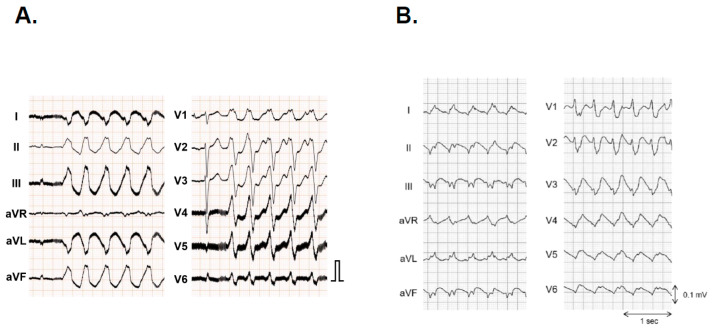
High-frequency noise on a surface electrocardiogram. (**A**) The surface electrocardiogram of a patient with an implanted HeartMate 3 during ventricular tachycardia showing high-frequency noise. (**B**) The surface electrocardiogram of an HVAD patient during ventricular tachycardia with no apparent noise.

Catheter ablation is widely recognized as a definitive treatment tool for tachyarrhythmias. Nevertheless, this technique presents particular challenges that elevate the risk of complications in LVAD support situations. To commence, in order to secure the success of procedures, the three-dimensional mapping systems that employ magnetic field sensors are of paramount importance. However, the issue of electromagnetic interference between three-dimensional mapping systems and LVAD motors is a significant concern in the context of precise electrophysiological studies. The previous devices, such as HVAD and HeartMate II, are less likely to interfere with magnet-based mapping systems. Nevertheless, there were some cases that exhibited limitations in visualizing the catheter’s position or performing electrophysiological mapping, particularly in the inferior or septal apical segments around the cannula and facing the turbine (Figure 2) [46]. HeartMate 3 is more likely to produce high-frequency noise on surface electrocardiograms, which may be mitigated through an ingenious adjustment of the low-pass filter settings (Figure 3) [47]. The incidence of electromagnetic interference was reported as 1.8% in the meta-analysis [25].

In patients with continuous-flow LVADs, it is common practice to employ invasive hemodynamic monitoring during the ablation procedures due to the inability to measure cuff pressure noninvasively. In the context of left heart access techniques, it is notable that the majority of ventricular tachyarrhythmias observed in LVAD recipients have their origin within the left ventricle. However, a trans-aortic valve approach is impractical due to the aortic transvalvular gradient, which promotes aortic valve closure [48]. A temporary reduction in pump speed may be effective in facilitating the opening of the aortic valve. In cases where a trans-septal approach is employed, the presence of a residual iatrogenic atrio-septal defect can occasionally result in a right-to-left shunt due to the strong suction of the LVAD, leading to hypoxia [49]. While a case report from Japan demonstrated the iatrogenic persistent atrio-septal defect following catheter ablation, measuring 2 mm in size, spontaneously occluded over time, transcatheter closure should be considered in situations of unacceptable hypoxia or right ventricular dysfunction [49,50]. The insertion of a single 8.5Fr (2.8 mm) steerable sheath, a widely used option, represents the greatest available method for preventing a residual defect, and follow-up echocardiograms after the procedure are imperative.

While LVAD recipients are typically prescribed warfarin anticoagulant therapy with a target international normalized ratio level of <3.5, intravenous heparin should be administered to achieve an activated clotting time exceeding 300 s when accessing the left atrial or ventricular endocardium [51]. Therefore, vascular access-related hemorrhage or bleeding is a significant complication in LVAD recipients, and in some cases, it necessitates a blood transfusion [52]. Ultrasound-guided vascular puncture and the use of vascular closure devices (Perclose ProStyle, Abbott, Chicago, IL, USA) or figure-of-8 sutures may be effective in reducing these complications.

An observational study reported an increased risk of pump thrombosis and thromboembolic events following endocardial ablation [53]. Hemolysis is one of the major complications in patients with durable LVADs; however, there is limited information available in previous registries [52,54]. While catheter entrapment by the cannula is a significant concern when performing catheter ablation for LVAD recipients, there have been no reports of this complication [25].

These procedural complications were reported to have occurred in 9.4% of ablation cases among LVAD recipients, a rate higher than that observed in patients without LVAD implantation [25,55]. The subxiphoid puncture technique for the epicardial approach has been established as a safe method in cases of VT originating from the epicardium. However, the presence of adhesions within the space is easily foreseeable in LVAD recipients, posing a significant challenge following LVAD implantation. Pre-implantation ablation may prove useful for postoperative suppression, and we eagerly await the results of the forthcoming clinical trial [56]. Essentials for tachyarrhythmia management with catheter ablation in LVAD recipients are summarized in Table 1.

### 3.2. For Atrial Tachyarrhythmias

AF is the predominant supraventricular arrhythmia in patients with LVADs [57]. However, as outlined in the above section, AF appears to have a lesser impact on the development of adverse prognostic events. Furthermore, attention should be paid to iatrogenic atrio-septal defects resulting from AF ablation. Consequently, the consideration of AF ablation for LVAD recipients should be approached with caution when compared to heart failure patients without LVAD support. Atrioventricular nodal ablation may be an effective option for rate control in patients with cardiac implantable pacing devices refractory to drug therapies, with attention to the absence of atrial kick resulting in unfavorable effects for hemodynamics like in Figure 1.

Atrial flutter, occurring in up to 50% of patients after open-heart surgery and 15% of patients following LVAD implantation, represents another significant form of atrial tachyarrhythmia [57,58]. Similar to AF, sustained atrial flutter leads to the loss of atrioventricular synchrony and right ventricular failure [59]. Unlike AF, typical atrial flutter is dependent on the tricuspid annulus, and trans-septal access for ablation is not required. Given the previous studies that have demonstrated a high success rate in LVAD recipients, catheter ablation for atrial flutter should be regarded as the primary therapy [59].

### 3.3. For Ventricular Tachyarrhythmias

In cases of recurrent VT presenting with complaints of palpitations or hemodynamic compromise manifesting as low-flow alerts, VT ablation should be given due consideration [38]. Over 90% of VTs are associated with reentry phenomena linked to scarred regions resulting from underlying cardiac pathologies, as opposed to their occurrence in the proximity of the inflow cannula, which accounts for less than 20% [25]. Bundle-branch reentry should also be contemplated, as many cases exhibit conduction system impairment akin to that observed in the working myocardium [60]. For the diagnosis of tachyarrhythmias pertaining to the conduction system, the induction of VT from the atrium or the recording of the His-bundle for entrainment during tachycardia should be considered. The recurrence rate during follow-up subsequent to VT ablation has been reported to range from 15% to 86% [47]. In a patient with a continuous-flow pump, the reappearance of VT should trigger an evaluation to exclude a suction event. The angle of the inflow cannula of the HeartMate II (Abbott Labs, Chicago, IL, USA) may exert an influence on the occurrence of suction-induced VT [61]. The HeartMate 3, equipped with a shorter inflow cannula compared to the HeartMate II, may result in a reduction of such events.

**Figure 4:**
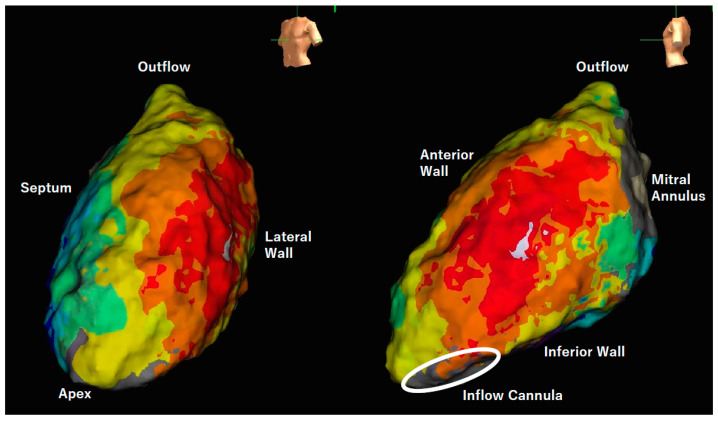
Activation map of ventricular tachycardia in a patient with HeartMate 3 support.

The short-term procedural success rate for VT ablation has been reported to be approximately 80% [25]. The major reasons for recurrence following endocardial ablation were VT origins located in the intramural or epicardial regions (Figure 4). Pre-implantation ablation or surgical ablation concomitant with the initial LVAD implantation may offer benefits for VTs originating deep within the myocardium. Chemical ablation through coronary venous branches with a double-balloon approach, cardiac stereotactic radioablation, or surgical ablation may serve as valuable approaches for refractory cases [39,62,63,64]. Autonomic nervous modulation, as exemplified by the stellate ganglion block, is also effective in suppressing electrical storms [65].

The activation map within the left ventricle revealed centrifugal propagation originating from the lateral wall, with the white area indicating the earliest activation site. This arrhythmia could not be terminated by radiofrequency delivery due to the speculated origin within the intramural layer or epicardium. The surface electrocardiogram of the present tachycardia is shown in Figure 3A.

VF is generally a formidable challenge to address via catheter ablation. Nonetheless, directing efforts towards addressing initiated ventricular premature contractions may prove effective in diminishing the recurrence of VF, particularly in cases of intractable electrical storms accompanied by hemodynamic compromise [37]. It is worth noting that there have been reports of three cases involving prolonged VF episodes in patients under mechanical circulatory support, where catheter ablation was focused on fragmented potentials exhibiting high frequency during VF, resulting in the spontaneous termination of VF [66]. The effects of the distinctive technique in LVAD recipients known as “de-networking”, which targets the Purkinje network, remain relatively obscure [67].

## 4. Significance of Managing across Multiple Professions

Patients supported with LVADs demonstrate diverse adverse events such as right ventricular failure, an increase in pulmonary vascular resistance, hematic congestion, as well as an increased burden of arrhythmias. These events are not manifested in isolation; rather, they are intricately interconnected. Effective management of these events necessitates a multifaceted approach, encompassing not only arrhythmia treatment but also adjustments in medication and optimization of LVAD speed by circulatory support specialists [68]. In addition, surgeons play a pivotal role in the management of refractory arrhythmias following LVAD implantation. Video-assisted thoracoscopic sympathectomy might be an option for suppressing recurrent VT refractory to catheter ablation [69].

## 5. Conclusions

**Figure 5:**
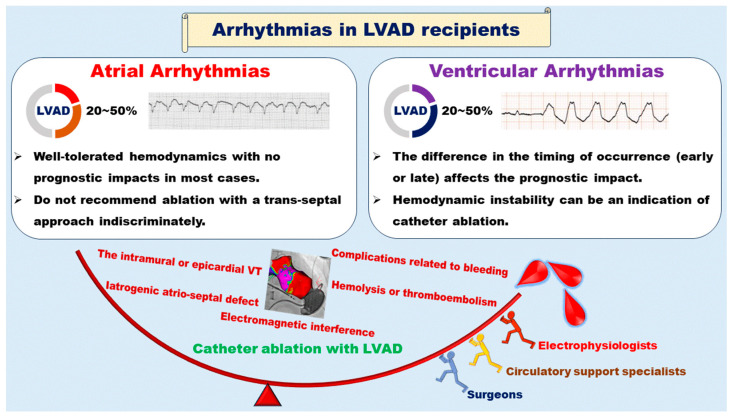
Illustrated summary of arrhythmia management.

Tachyarrhythmias originating from both the atrium and the ventricle are frequently encountered in LVAD recipients. While robust hemodynamic support typically mitigates the prognostic impact of tachyarrhythmias, some cases still result in a decline in their quality of life or hemodynamic compromise, necessitating invasive catheter ablation. Due to the distinctive hemodynamics and patient vulnerability inherent in LVAD recipients, catheter ablation-related complications exhibit a wide spectrum of diversity, rendering them more susceptible to occurrence compared to patients without LVAD implantation. The population of LVAD-supported patients is expected to increase, serving as both a bridge to transplant therapy and a destination therapy. To address the intricate and refractory arrhythmia issues in LVAD patients, not only electrophysiologists but also circulatory support specialists and surgeons should collaborate with each other. There is also a pressing need for further accumulation of knowledge concerning the impacts of catheter ablation and the associated procedural complications (see Figure 5).

The prognostic impacts of arrhythmias differ between atrial and ventricular arrhythmias. Several issues related to catheter ablation should be managed by specialists in electrophysiology, circulatory support, and surgery.

## Figures and Tables

**Table 1:** Essentials for tachyarrhythmia management with catheter ablation in LVAD recipients.

	Atrial Tachyarrhythmias	Ventricular Tachyarrhythmias
The key factors for making the decision to perform catheter ablation		
Hemodynamic instability	✓ Most cases are tolerable.	✓ Some cases exhibit a low-flow alert, with or without palpitations.
Prognostic impacts	✓ Fewer impacts on the prognosis.	✓ Early onset (within <30 days after implantation) may have different impacts compared to those of late onset.
Types of arrhythmias for which catheter ablation is recommended	✓ Atrial flutter	✓ Sustained ventricular tachycardia, especially in the state of electrical storm.
Complications		
Electromagnetic interference	✓ N/A	✓ The area around the inflow cannula at the apex.
Iatrogenic atrio-septal defect	✓ Atrioventricular nodal ablation is one of the options for avoiding this complication.	✓ Insert a single sheath through the septal puncture and prepare for transcatheter closure if necessary.
Hemolysis or thromboembolism	N/A	✓ Exercise caution, especially during ablation around the inflow cannula.
How to perform		
Preprocedural imaging	✓ Cardiac computed tomography contributes to understanding the three-dimensional spatial relationship.	
Approaches for the left heart	✓ Trans-septal approach (Impractical trans-aortic valve approach due to valve closure).	
Cases of intramural or epicardial origin	✓ Difficulties with the subxiphoid puncture. ✓ Chemical ablation, cardiac stereotactic radioablation, or surgical ablation may have value.	
Intracardiac echocardiography	✓ In cases where there is an area of interest in the left ventricle, viewing the catheter from the right ventricle is valuable for avoiding interference with the inflow cannula.	

## Data Availability

Not applicable.

## Abbreviations

AF	atrial fibrillation
LVAD	left ventricular assist device
VT	ventricular tachycardia
VF	ventricular fibrillation
